# Dynamic effects of black soldier fly larvae meal on the cecal bacterial microbiota and prevalence of selected antimicrobial resistant determinants in broiler chickens

**DOI:** 10.1186/s42523-024-00293-9

**Published:** 2024-02-15

**Authors:** Calvin Ho-Fung Lau, Sabrina Capitani, Yuan-Ching Tien, Lou Ann Verellen, Munene Kithama, Hellen Kang, Elijah G. Kiarie, Edward Topp, Moussa S. Diarra, Michael Fruci

**Affiliations:** 1 Ottawa Laboratory (Carling), Canadian Food Inspection Agency, Ottawa, ON Canada; 2 London Research and Development Centre, Agriculture and Agri-Food Canada, London, ON Canada; 3 Guelph Research and Development Centre, Agriculture and Agri-Food Canada, Guelph, ON Canada; 4 Department of Animal Biosciences, University of Guelph, Guelph, ON Canada; 5 Present Address: School of Medicine, Faculty of Health Sciences, Queen’s University, Kingston, ON Canada; 6 Present Address: Agroécologie research unit, INRAE, Université de Bourgogne, Dijon, France; 7 Department of Microbiology and Immunology, University of Western Ontario, London, ON Canada

**Keywords:** Black soldier fly larvae, Poultry, Cecal bacterial microbiota, Antimicrobial resistance

## Abstract

**Background:**

We had earlier described the growth-promoting and -depressive effects of replacing soybean meal (SBM) with low (12.5% and 25%) and high (50% and 100%) inclusion levels of black soldier fly larvae meal (BSFLM), respectively, in Ross x Ross 708 broiler chicken diets. Herein, using 16S rRNA gene amplicon sequencing, we investigated the effects of replacing SBM with increasing inclusion levels (0-100%) of BSFLM in broiler diets on the cecal bacterial community composition at each growth phase compared to broilers fed a basal corn-SBM diet with or without the in-feed antibiotic, bacitracin methylene disalicylate (BMD). We also evaluated the impact of low (12.5% and 25%) inclusion levels of BSFLM (LIL-BSFLM) on the prevalence of selected antimicrobial resistance genes (ARGs) in litter and cecal samples from 35-day-old birds.

**Results:**

Compared to a conventional SBM-based broiler chicken diet, high (50 to100%) inclusion levels of BSFLM (HIL-BSFLM) significantly altered the cecal bacterial composition and structure, whereas LIL-BSFLM had a minimal effect. Differential abundance analysis further revealed that the ceca of birds fed 100% BSFLM consistently harbored a ~ 3 log-fold higher abundance of *Romboutsia* and a ~ 2 log-fold lower abundance of *Shuttleworthia* relative to those fed a BMD-supplemented control diet at all growth phases. Transient changes in the abundance of several potentially significant bacterial genera, primarily belonging to the class *Clostridia*, were also observed for birds fed HIL-BSFLM. At the finisher phase, *Enterococci* bacteria were enriched in the ceca of chickens raised without antibiotic, regardless of the level of dietary BSFLM. Additionally, bacitracin (*bcrR*) and macrolide (*ermB*) resistance genes were found to be less abundant in the ceca of chickens fed antibiotic-free diets, including either a corn-SBM or LIL-BSFLM diet.

**Conclusions:**

Chickens fed a HIL-BSFLM presented with an imbalanced gut bacterial microbiota profile, which may be linked to the previously reported growth-depressing effects of a BSFLM diet. In contrast, LIL-BSFLM had a minimal effect on the composition of the cecal bacterial microbiota and did not enrich for selected ARGs. Thus, substitution of SBM with low levels of BSFLM in broiler diets could be a promising alternative to the antibiotic growth promoter, BMD, with the added-value of not enriching for bacitracin- and macrolide-associated ARGs.

**Supplementary Information:**

The online version contains supplementary material available at 10.1186/s42523-024-00293-9.

## Introduction

Global consumption of meat protein is expected to increase over the next decade, mainly due to increasing population growth and dietary changes associated with growing affluence [1]. Currently, poultry meat comprises 59% of total global meat production and is expected to rise to 62% by the year 2032, largely driven by growing consumer demand[1, 2]. This demand will undoubtedly be met with an increase in production of poultry feed, which primarily consists of corn and soybean meal (SBM) [1]. SBM is routinely added to poultry feed as the main source of crude protein and amino acids [3]. However, soybean production and its supply chain are associated with negative environmental consequences, including deforestation, biodiversity loss, pesticide use, soil depletion, water usage, and greenhouse gas emissions [4–8]. In addition to these environmental costs, there is also a growing global health concern regarding in-feed antibiotic use in poultry diets for growth promotion and prophylactic purposes, as the inappropriate use of antibiotics has been linked to promoting the emergence and dissemination of antibiotic-resistant bacteria and antibiotic resistance genes (ARGs) that threaten modern medicine [9–11]. Faced with the double burden of finding sustainable protein feed and the growing restrictions and bans on antibiotic use in poultry production, it is crucial to identify and evaluate alternative feedstuffs with functional properties to produce poultry meat sustainably and profitably.

An emerging and novel sustainable protein alternative to SBM in poultry feed is meal prepared from the larvae of black soldier flies [*Hermetia illucens* (Diptera: Stratiomyidae)]. Black soldier fly larvae are capable of efficiently upcycling food wastes and organic wastes such as fruits, vegetables, and manure into a high-quality proteinaceous feed [12–14]. Apart from this, black soldier fly larvae are also a source of antimicrobial and immunomodulatory compounds such as chitin [15], lauric acid [16], and antimicrobial peptides [17]. Focusing on its potential as a substitute ingredient of conventional poultry feeds, we have recently shown that replacement of SBM with low inclusion levels of black soldier fly larvae meal (LIL-BSFLM; defined here as 12.5% and 25%) can provide for a positive growth response in broiler chickens during the starter period of growth, comparable to those fed a conventional corn-SBM-based diet supplemented with the antibiotic growth promoter, bacitracin methylene disalicylate (BMD) [18]. Likewise, Facey et al. demonstrated that substituting SBM in broiler chicken diets with 12.5% and 25% BSFLM fortified with BSFLM oil also had performance-enhancing effects similar to a conventional SBM-based diet supplemented with in-feed BMD and narasin during the starter phase [19]. Conversely, we observed that high inclusion levels of BSFLM (HIL-BSFLM, defined here as 50% and 100% BSFLM) in broiler chicken diets had a detrimental impact on the overall growth performance of broiler chickens, a finding that had also been reported by several other studies [18–21].

In recent years, the intestinal microbiota of poultry has received considerable attention as it plays an important role in various processes that affect the overall health, growth, and performance of poultry. These processes include nutrient digestion and absorption [22], nitrogen recycling by metabolising urea [23], supplying B vitamins to their hosts [24], production of essential amino acids [24], modulation of the immune system [25], and colonization resistance to enteric pathogens [26]. Within the intestinal tract, the twin ceca (located where the small and large intestines meet) contains the largest number and diversity of bacteria (10^11^-10^12^ colony forming units/g) [27]. It has been suggested that a well-functioning cecum is responsible for providing 10% of the energy needs for chickens, as it is an important region for microbial fermentation of non-starch polysaccharides and the subsequent production of short chain fatty acids (SCFAs) [28, 29]. Additionally, the chicken cecal microbiota also acts as a major reservoir of chicken and zoonotic pathogens, as well as antimicrobial resistance determinants [30]. Thus, the cecal microbiota and their associated metabolites have a profound influence on health and disease and is increasingly regarded as a potential modifiable risk factor and therapeutic target to promote broiler health and performance [31]. Herein, to gain a deeper understanding of the chicken intestinal microbiome and its dynamics in response to dietary BSFLM, we analyzed the bacterial composition of the cecal microbiota of broilers fed increasing inclusion levels of BSFLM (0-100%) in search of any potential links between gut bacteria and the BSFLM-mediated growth promotion and depression. In the context of antimicrobial resistance mitigation, we also examined the impact of performance-enhancing amounts of dietary BSFLM on selected antimicrobial resistance gene (ARG) abundance in chicken cecal and litter samples with the purpose of providing insight into a potential value-added benefit of using BSFLM as an alternative feed ingredient.

## Results

### Impact of diet on the development of the core cecal bacterial microbiota of broiler chickens

A total of 288 cecal samples were collected (1 ceca x 2 birds per replicate x 8 replicates x 6 diets x 3 sampling times). Of these, twenty samples were excluded from analyses due to a lack of digesta present in the cecum, and four samples were excluded after 16S gene amplicon sequencing and quality filtering. Therefore, a total of 264 cecal samples were included in the 16S rRNA gene amplicon analysis. The detailed distribution of cecal samples analyzed for each diet, including the respective sample sizes (n) for days 14, 28 and 35, were as follows: BMD (*n* = 14, 15, 15), 0% BSFLM (*n* = 13, 16,15), 12.5% BSFLM (*n* = 12, 15, 16), 25% BSFLM (*n* = 14, 16, 16), 50% BSFLM (*n* = 14, 16, 15), and 100% BSFLM (*n* = 14, 16, 16).

**Fig. 1:**
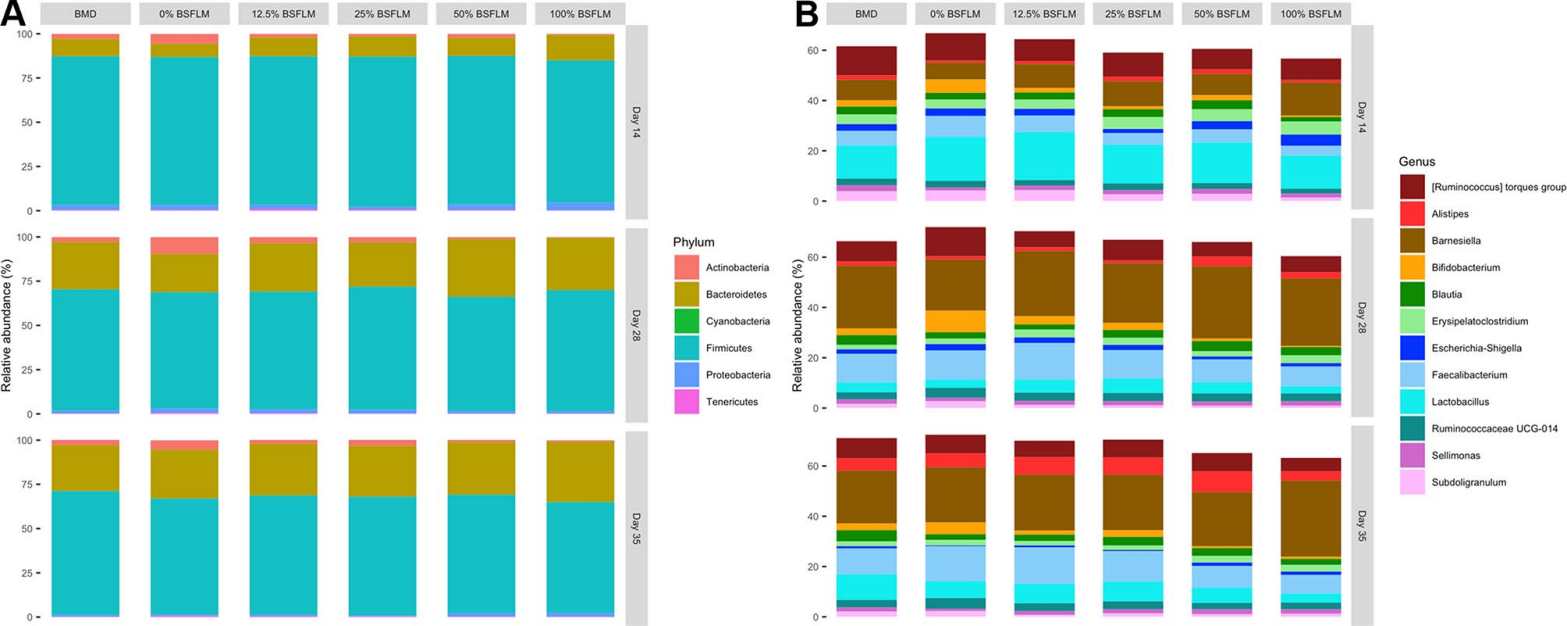
Taxonomic composition of the broiler chicken cecal bacterial communities at the phylum (**A**) and genus (**B**) level. Data are grouped by phase of growth (Day 14, 28 and 35) and diet (BMD, 0%, 12.5%, 25%, 50% and 100% BSFLM), with the results presented as averaged values obtained from up to sixteen individual biological replicates per group

By profiling the bacterial composition of over 260 cecal samples collected from broiler chickens subjected to conventional corn-SBM-based diets and the BSFLM-substitution diets throughout a three-stage production cycle (see Additional file 1, Table S1, for 16S rRNA gene sequencing metrics), it was consistently observed that *Firmicutes* (72.33% ± 0.21%, mean ± SEM) and *Bacteroidetes* (22.24% ± 0.22%) remained to be the predominant phyla in ceca spanning all three growth phases (Fig. 1A). At the genus level, a shift in the chicken cecal bacterial composition that was driven by age was revealed. For instance, at the end of the starter phase (day 14), *Lactobacillus* had the highest overall abundance and comprised an estimated 13.20-19.08% of the total bacterial content of the ceca, whereas *Barnesiella* was the most prevalent genus detected in birds sampled at later stages of growth (i.e., grower and finisher phases) (Fig. 1B). There was also a noticeable expansion of *Alistipes* bacteria in the ceca of 35-day-old chickens compared to those of the younger cohorts (Fig. 1B). The partial/complete replacement of SBM with BSFLM in chicken diets appeared to have little influence on the age-dependent dynamics of the core bacterial constituents of the boiler’s cecal bacterial microbiota, illustrating the stability and resilience of the chicken gut microbiome to dietary changes.

### Impact of diet on the bacterial diversity of the broiler chicken cecum

**Fig. 2:**
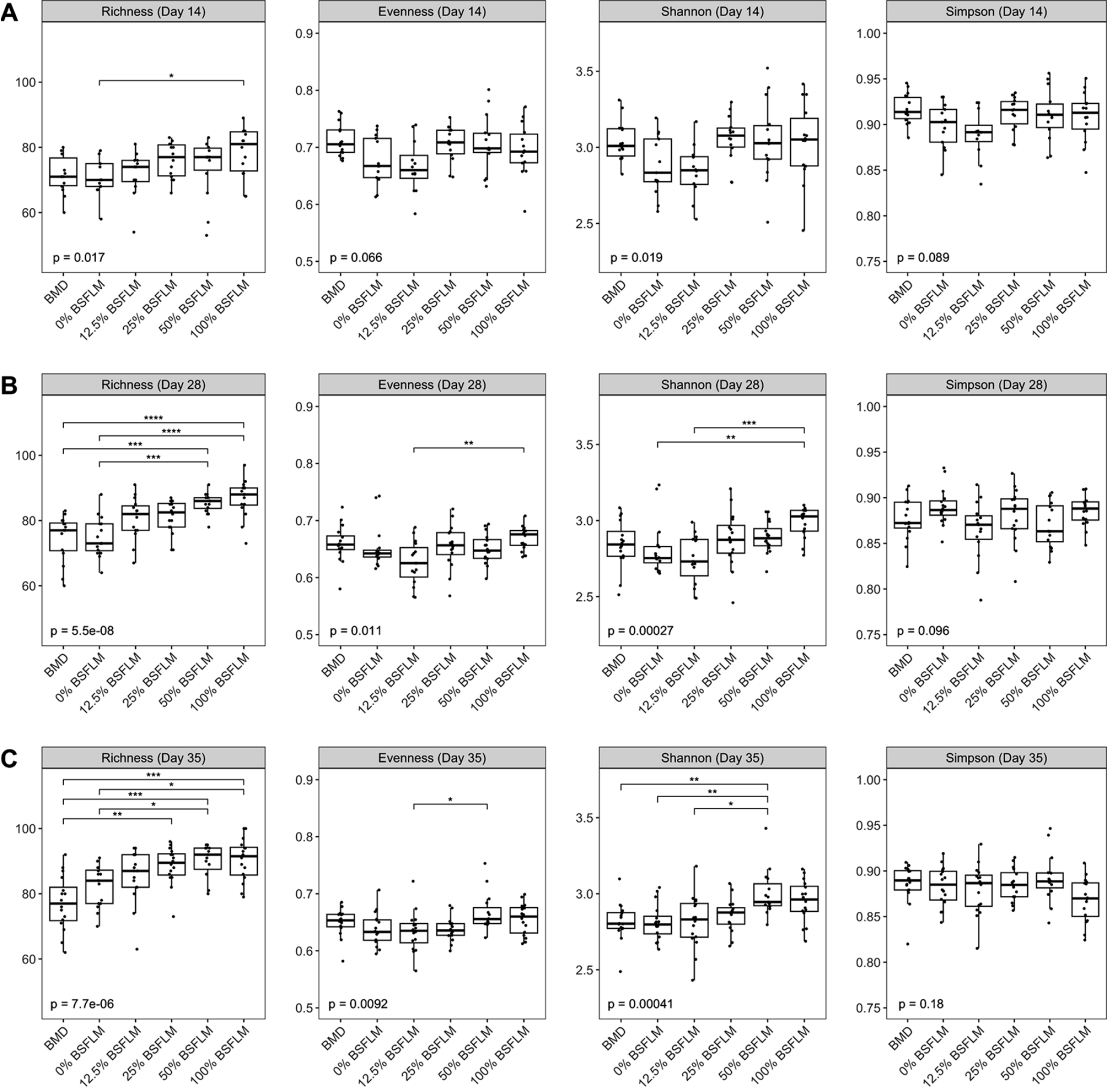
Alpha diversity metrics of the cecal bacterial community recovered from 14- (**A**), 28- (**B**), and 35-day-old (**C**) broiler chickens fed varied amounts of BSFLM and a conventional soybean-based diet with or without BMD. The box-and-whisker plots show the first and third quartile (bottom and top lines of the box) and the median (horizontal middle line) values of the indicated diversity metrics. Each broiler chicken cecal sample is represented by a solid black dot. Statistical significance of between-group differences were detected using the non-parametric Kruskal-Wallis test followed by post-hoc pairwise Dunn test with Bonferroni correction and are indicated with an asterisk as follows: * *p* < 0.05; ** *p* < 0.01; *** *p* < 0.001; **** *p* < 0.0001. BMD, bacitracin methylene disalicylate; BSFLM, black soldier fly larvae meal; %, percentage of soybean meal replaced with black soldier fly larvae meal (BSFLM)

To better explore and compare the cecal bacterial community structure of broiler chickens raised on different diets, alpha-diversity indices including taxon richness, Shannon and Simpson indices, and Pielou’s evenness were estimated using genus-level taxonomic data. On day 14, no significant difference between dietary groups were detected for any of the computed diversity metrics, with the exception that a significant increase in bacterial richness observed for chickens fed 100% BSFLM relative to those in the 0% BSFLM group (*p* < 0.05) (Fig. 2A). On day 28 and 35, both the 50% and 100% BSFLM groups displayed a significantly higher level of bacterial richness relative to those receiving a SBM-based diet with (i.e., BMD group) and without (i.e., 0% BSFLM group) supplementation with the antibiotic growth promoter, BMD (*p* < 0.05) (Fig. 2B and C), although that did not coincide with any statistically meaningful differences in both evenness and Simpson’s diversity index. Shannon’s index, on the contrary, was sensitive to the BSFLM content of the chicken diets on day 28 and 35, with significant diversity variance being detected mainly between high and low BSFLM-content diet groups (Fig. 2B and C).

While there was a general lack of difference in all four alpha-diversity indicators between the cecal bacterial communities derived from the BMD and 0% BSFLM diet groups throughout the duration of a 5-week sampling period (Fig. 2), bacterial richness was observed to significantly increase overtime only for the broiler chicken populations without dietary exposure to BMD (i.e., the 0% BSFLM group)(see Additional File 2, Figure S1A and S1B). This age-dependent increase in bacterial richness was also conserved among the other four antibiotic-free BSFLM diet groups (see Additional file 2, Figure S1C-F). Together these data suggest that BMD abrogates the developmental expansion of bacterial community richness in the broiler’s cecum.

Based on the principal coordinate analysis (PCoA) performed using Bray-Curtis’s distance matrices and the corresponding PERMANOVA statistics (see Additional file 2, Figures S2 and S3), the observed variance in the chicken gut microbial community between individual groups can be explained by differences in both age (Figure S2) and diet (Figure S3). No significant interaction of these two explanatory variables was detected (see Additional file 1, Table S2), implying the age- and diet-factors likely contribute independently to the microbiome dissimilarities. As indicated by the error-corrected multiple pairwise comparisons (Table 1), the bacterial composition of the ceca of 14-day-old broiler chickens fed 100% BSFLM differed significantly from the BMD (*p* < 0.015), 0% BSFLM (*p* < 0.015) and 12.5% BSFLM (*p* < 0.030) groups. On day 28, the bacterial composition significantly differed between the majority of diets, with the exceptions being among the BMD, 12.5%, and 25% BSFLM groups and between the 50% and 100% BSFLM groups (Table 1). Finally, at the finisher-phase, broiler chickens fed 0%, 12.5%, and 25% BSFLM diets and the conventional antibiotic-supplemented SBM-based diet (BMD group), exhibited a more similar cecal bacterial composition than broiler chickens fed HIL-BSFLM (i.e., 50–100% BSFLM) (Table 1). Taken together, these data demonstrate that HIL-BSFLM, particularly at 100% BSFLM, results in significant changes in the composition of the cecal microbiota, whereas LIL-BSFLM have only a minimal effect.

**Table 1:** Multiple pairwise comparisons of the cecal bacterial microbiota between broiler chickens fed varied amounts of BSFLM and a conventional soybean-based diet with or without BMD

		Pairwise comparison statistics^2^	
**Day**	**Diet Comparisons** ^**1**^	R^2^ value	Adjusted *p* value^**3**^
14			
	BMD vs. 0% BSFLM	0.0462	1.000
	BMD vs. 12.5% BSFLM	0.0394	1.000
	BMD vs. 25% BSFLM	0.0539	1.000
	BMD vs. 50% BSFLM	0.0398	1.000
	BMD vs. 100% BSFLM	0.1402	**0.015**
	0% vs. 12.5% BSFLM	0.0377	1.000
	0% vs. 25% BSFLM	0.0835	0.270
	0% vs. 50% BSFLM	0.0651	1.000
	0% vs. 100% BSFLM	0.189	**0.015**
	12.5% vs. 25% BSFLM	0.0379	1.000
	12.5% vs. 50% BSFLM	0.0366	1.000
	12.5% vs. 100% BSFLM	0.1365	**0.030**
	25% vs. 50% BSFLM	0.0227	1.000
	25% vs. 100% BSFLM	0.0888	0.180
	50% vs. 100% BSFLM	0.0682	0.840
28			
	BMD vs. 0% BSFLM	0.1446	**0.015**
	BMD vs. 12.5% BSFLM	0.0472	1.000
	BMD vs. 25% BSFLM	0.0293	1.000
	BMD vs. 50% BSFLM	0.0827	**0.045**
	BMD vs. 100% BSFLM	0.1243	**0.015**
	0% vs. 12.5% BSFLM	0.1509	**0.015**
	0% vs. 25% BSFLM	0.1358	**0.015**
	0% vs. 50% BSFLM	0.2988	**0.015**
	0% vs. 100% BSFLM	0.3445	**0.015**
	12.5% vs. 25% BSFLM	0.0473	1.000
	12.5% vs. 50% BSFLM	0.1312	**0.015**
	12.5% vs. 100% BSFLM	0.1862	**0.015**
	25% vs. 50% BSFLM	0.0809	**0.030**
	25% vs. 100% BSFLM	0.1270	**0.015**
	50% vs. 100% BSFLM	0.0823	0.075
35			
	BMD vs. 0% BSFLM	0.0581	1.000
	BMD vs. 12.5% BSFLM	0.0810	0.300
	BMD vs. 25% BSFLM	0.0558	1.000
	BMD vs. 50% BSFLM	0.1225	**0.030**
	BMD vs. 100% BSFLM	0.2998	**0.015**
	0% vs. 12.5% BSFLM	0.0509	1.0000
	0% vs. 25% BSFLM	0.0609	0.855
	0% vs. 50% BSFLM	0.1680	**0.015**
	0% vs. 100% BSFLM	0.3223	**0.015**
	12.5% vs. 25% BSFLM	0.0320	1.0000
	12.5% vs. 50% BSFLM	0.1008	0.075
	12.5% vs. 100% BSFLM	0.2665	**0.015**
	25% vs. 50% BSFLM	0.0971	0.060
	25% vs. 100% BSFLM	0.2744	**0.015**
	50% vs. 100% BSFLM	0.1459	**0.015**

^1^ BMD, bacitracin methylene disalicylate control; BSFLM, black soldier fly meal; %, percentage of soybean meal replaced

with black soldier fly larvae meal (BSFLM)

^2^Error-corrected multiple pairwise comparison was performed using R package *pairwiseAdonis* with Bonferroni correction

and 999 permutations

^3^Significant differences are bolded (*p* < 0.05)

### Impact of BSFLM diet on the abundance of individual bacterial taxa in the broiler chicken cecum

**Fig. 3:**
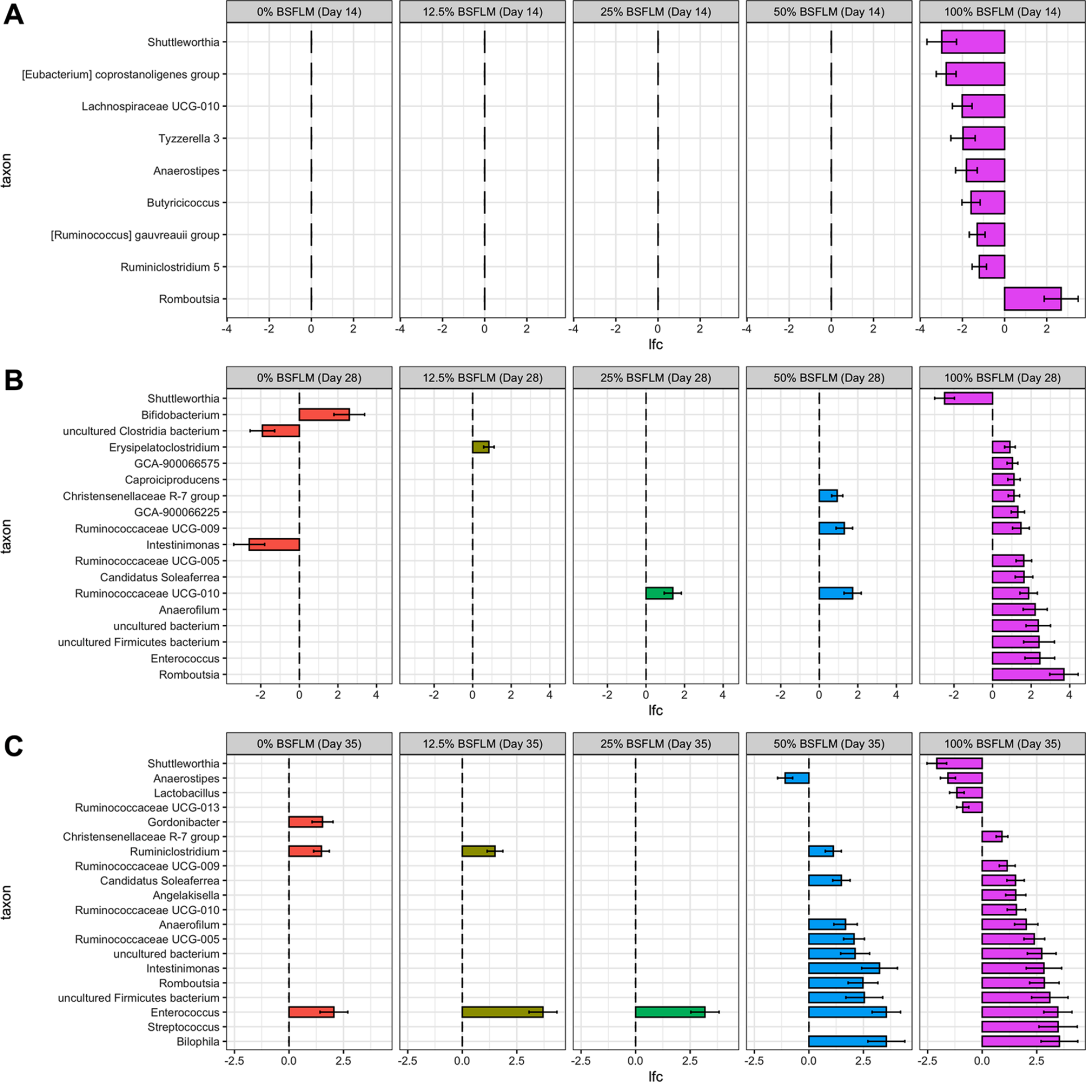
Cecal bacterial genera significantly affected by the dietary exposure to different levels of BSFLM on day 14 (**A**), day 28 (**B**), and day 35 (**C**). Horizontal bars represent log fold change ± standard error (shown as error bars) of absolute abundance associated with 0%, 12.5%, 25%, 50%, or 100% BSFLM diets relative to the BMD diet (reference) as determined by differential abundance analysis performed using ANCOM-BC2. Only taxa with statistically significant changes in their abundance relative to the reference group were displayed

To further characterize the changes in the bacterial community structure associated with dietary exposure to various levels of BSFLM at different ages, differential abundance analysis was conducted using ANCOM-BC [32] to identify key bacterial taxa whose absolute abundance was impacted by the BSFLM-based diets relative to the BMD control diet. On day 14, no significant differences in the differential abundance of taxa at the genus level were detected for any of the 050% BSFLM groups compared to the BMD control (Fig. 3A). In contrast, birds fed 100% BSFLM exhibited a significantly lower abundance of bacteria belonging to the genera *Shuttleworthia*, [Eubacterium] *coprostanoligenes* group, *Lachnospiraceae* UCG-010, *Tyzzerella* 3, *Anaerostipes*, *Butyricicoccus*, [*Ruminococcus*] *gauvreauii* group and *Ruminiclostridium* 5 (Fig. 3A). In addition, there was a greater than 2 log-fold increase in the absolute abundance of *Romboutsia* bacteria in birds fed 100% BSFLM relative to the BMD-fed birds.

On day 28, a higher level of *Bifidobacterium* and lower abundance of uncultured *Clostridia* bacterium and *Intestinimonas* were associated with birds fed 0% BSFLM (i.e., absence of both BMD and BSFLM) (Fig. 3B). The abundance of only a few bacterial genera were altered for chickens fed 12.5%, 25%, and 50% BSFLM diets relative to the BMD control (Fig. 3B). For example, *Erysipelatoclostridium* was found to be more abundant in birds fed 12.5% BSFLM, while both *Christensenellaceae R-7* group, and *Ruminococcaceae* UCG-009 bacteria had greater abundance in the 50% BSFLM group (Fig. 3B). For birds receiving a 100% BSFLM diet, a total of 14 genera, including *Romboutsia* (also identified on day 14), and *Ruminococcaceae* UCG-010, which was also commonly observed in the 25% and 50% BSFLM groups, displayed significant increases in their absolute abundance relative to BMD-fed birds (Fig. 3B). Like day 14, *Shuttleworthia* was less abundant in the 100% BSFLM group compared to BMD, on day 28.

On day 35, *Enterococcus* bacteria were more abundant in the ceca of chickens fed a non-BMD diet, regardless of the BSFLM percentage content (Fig. 3C). Intriguingly, birds that received 50% and 100% BSFLM displayed several similar changes in the abundance of specific bacterial genera when compared to those fed the BMD control diet. Excluding the genera *Enterococcus* and *Ruminiclostridium*, whose elevated abundance appeared to be a common response to a non-BMD diet rather than a BSFLM-dependent one, *Candidatus Soleaferrea, Anaerofilum*, *Ruminococcaceae* UCG-005, *Intestinimonas, Romboutsia, Bilophila*, together with two uncultured genera, were all found to be more abundant in absolute-terms among both the 50% and 100% BSFLM diet groups (Fig. 3C). Several additional genera were observed to increase in abundance in the 100% BSFLM group and included *Streptococcus*, *Ruminococcaceae* UCG-010, *Angelakisella*, *Ruminococcaceae* UCG-009, and *Christensenellaceae* R-7 group (Fig. 3C). On the contrary, in addition to the *Shuttleworthia* population that had remained diminished since day 14, the abundance of bacteria belonging to the genera *Anaerostipes, Lactobacillus,* and *Ruminicoccaceae* UCG-013 were also reduced in the 100% BSFLM group (Fig. 3C).

### Effect of diet on antibiotic resistance gene abundance in broiler chicken ceca and litter

Previously, we had demonstrated that replacement of SBM in broiler chicken diets with LIL-BSFLM had some growth performance-enhancing properties comparable to chickens raised on a conventional SBM-based diet supplemented with BMD [33]. To further evaluate whether substitution of SBM with BSFLM could influence the levels of antimicrobial resistance (AMR) determinants within the poultry production system, quantitative real-time PCR assays were performed on both cecal and litter DNA samples taken from 35-day-old chickens and the abundance of fourteen AMR-associated gene targets were measured. According to the normalized gene quantification data derived from the litter samples collected, no significant differences in the relative abundance of all gene targets evaluated were observed between chickens that received BMD and 0, 12.5, or 25% BSFLM diets (Table 2). However, the bacitracin resistance gene, *bcrR*, and the macrolide resistance gene, *ermB*, were found to be less abundant in the ceca of chickens following the 0%, 12.5% and 25% BSFLM dietary regime relative to those fed the BMD supplemented diet (Table 2). This observation suggests that elimination of BMD from conventional poultry feed can indeed diminish the abundance of bacitracin- and macrolide-associated resistance genes and that partial replacement of SBM with BSFLM has no significant effect on influencing the levels of selected gene targets within the chicken gut.

**Table 2:** The abundance of selected antimicrobial resistance determinants in day 35 chicken litter and cecal samples of birds fed BMD, 0%, 12.5%, and 25% BSFLM

Target gene copy number/*rrnS* gene copy number (x 10^− 6^) ± SEM^2^								
**Diet** ^**1**^	Litter				Cecum			
	BMD	0%	12.5%	25%	BMD	0%	12.5%	25%
**Gene Target**								
*strA*	154026 ± 80294	288021 ± 127212	448690 ± 193791	199109 ± 68265	357 ± 231	5445 ± 3377	28210 ± 26643	2219 ± 1682
*strB*	24579 ± 12467	45758 ± 20377	78221 ± 40171	31973 ± 12834	79.9 ± 49.24	1432 ± 870	6896 ± 6428	531 ± 373
*tetA*	81110 ± 25549	219589 ± 88024	153168 ± 65460	104049 ± 22118	2058 ± 411	5984 ± 1733	1724 ± 419	2005 ± 935
*blaCMY-2*	24927 ± 20655	7491 ± 2646	44986 ± 20659	10421 ± 4924	32.6 ± 16.96	11.2 ± 4.12	38.6 ± 11.17	14.1 ± 6.59
*sul1*	3499 ± 2059	6692 ± 3563	3410 ± 2362	5248 ± 1416	37.1 ± 31.87	347 ± 313	49.5 ± 38.68	19.0 ± 11.79
*sul2*	20400 ± 11986	12249 ± 4469	26566 ± 10196	23327 ± 9266	5.63 ± 2.70	564 ± 555	83.2 ± 47.23	3.74 ± 3.20
*ermF*	0.228 ± 0.121	0.128 ± 0.031	0.146 ± 0.039	0.128 ± 0.022	BDL	BDL	BDL	BDL
*mphE*	0.228 ± 0.12	0.140 ± 0.037	1.10 ± 0.957	0.128 ± 0.022	BDL	BDL	BDL	BDL
*Int1*	23599 ± 11567	21405 ± 6644	22718 ± 8062	25089 ± 8320	92.64 ± 71.20	2725.±2595	373 ± 177	57.1 ± 31.22
*blaOXA-20*	0.697 ± 0.540	336 ± 335	0.801 ± 0.535	0.709 ± 0.264	0.501 ± 0.123	0.384 ± 0.084	1.72 ± 0.926	0.351 ± 0.073
*msrE*	0.228 ± 0.121	0.464 ± 0.235	8.5 ± 8.35	0.141 ± 0.025	BDL	BDL	BDL	BDL
*ermB*	5310 ± 2697	16431 ± 6966	6944 ± 2711	2783 ± 816	191787 ± 29057	**104689 ± 22512** ^*^	**93115 ± 15602** ^*^	**64585 ± 9922** ^**^
*aadA*	1578 ± 974	2705 ± 1465	1732 ± 0.52	2498 ± 617	27.2 ± 23.36	323 ± 291	42.8 ± 28.77	16.7 ± 10.25
*bcrR*	15954 ± 6021	4660 ± 1173	8188 ± 3583	6015 ± 1485	11465 ± 3494	**2385 ± 893** ^*^	**2004 ± 1051** ^**^	**1218 ± 648** ^**^

^1^ BMD, bacitracin methylene disalicylate control; BSFLM, black soldier fly meal; %, percentage of soybean meal replaced with black soldier fly larvae meal (BSFLM)

^2^ Values are presented as means ± standard error of the mean of 8 biological replicates. Non-parametric Mann-Whitney U tests were conducted to assess significant differences between the BMD group and the 0%, 12.5%, or 25% BSFLM groups for each antibiotic resistance determinant. Significant differences are indicated in bold, and p-values are denoted as follows: *, *p* < 0.05, ** *p* < 0. 005. BDL, below detection limit

## Discussion

Earlier work had shown that replacement of SBM with LIL-BSFLM in broiler chicken diets resulted in growth-promoting effects similar to chickens fed a conventional corn-SBM-based diet supplemented with BMD [18, 19], whereas HIL-BSFLM substitution negatively impacted growth performance [19, 20]. In the present study, we demonstrated that the cecal bacterial communities of chickens fed low amounts of BSFLM remained comparable to those that received the BMD control diet throughout a three-phase growth cycle. This suggests that the growth-enhancing properties of BSFLM and those of the antibiotic growth promoter, BMD, can not be sufficiently distinguished based on genus-level variations in the cecal bacterial community composition. In stark contrast, replacement of SBM with high amounts of BSFLM in chicken diets induced a significant modification of the cecal bacterial structure, including increased community richness and Shannon’s diversity (Fig. 1) and long-term enrichment/depletion of specific bacterial genera (Fig. 3). Together these observations indicate that high amounts of BSFLM results in an imbalanced cecal bacterial microbiota (i.e., dysbiosis) that could be potentially disruptive to the optimal growth of broiler chickens.

In agreement with our observations associated with diets containing high amounts of BSFLM on the cecal bacterial diversity of male broiler chickens, similar studies examining the effect of complete replacement of SBM or fish meal with BSFLM in Lohmann Brown Classic laying hen [21] and ISA brown layer chick [34] diets, have also reported increased in bacterial richness and Shannon’s diversity index, together with significant changes in beta diversity of gut bacterial communities [34]. In contrast, for diets with low BSFLM content, Biasato et al. showed that replacement of SBM with 15% BSFLM (but not 5% and 10%) was associated with a reduction in Shannon’s diversity in the cecal microbiota of 35-day-old broiler chickens [35]. However, we observed no significant differences in any of the diversity metrics between the 12.5% BSFLM diet group and both BSFLM-free control groups (i.e., BMD and 0% BSFLM groups). Other related studies investigating a different approach of supplementing (instead of substituting) corn-SBM-based diets with lower (1–5%) inclusion levels of BSFLM in poultry diets have also provided some evidence of BSFLM-dependent changes in the cecal bacterial community diversity [36, 37]. Nonetheless, due to variations in experimental conditions such as bird species, BSFLM inclusion levels and source, sampling frequency, and diversity indices evaluated, comparing studies regarding the effect of BSFLM towards shaping the chicken microbiome remains challenging. Furthermore, despite the findings reported here and elsewhere, it is unclear if these changes in the cecal bacterial diversity of broiler chickens is driven directly by the introduction of BSFLM into the diet, and/or indirectly due to factors such as lower feed intake (i.e., related to changes in appetite) and other host-specific responses to a modified diet.

Notably, the two genera *Shuttleworthia* and *Romboutsia* were consistently suppressed or enriched, respectively, in birds receiving 100% BSFLM at all phases of growth (Fig. 3). Previous studies have shown that the abundance of *Shuttleworthia* in the cecum of chickens positively correlates with body weight gain [38], body weight [39], average daily feed intake, average daily gain, and negatively correlates with the feed-to-gain ratio [40]. It is tempting to speculate, then, that depletion of this genus in birds receiving 100% BSFLM contributes to the poor performance observed in this group. In addition to depletion of *Shuttleworthia*, a temporary reduction in the genera *Eubacterium coprostanoligenes group*, *Lachnospiraceae* UCG-010, *Tyzerella 3*, *Anaerostipes*, *Butyricicoccus*, *Ruminococcus gauvreauii group*, and *Ruminiclostridium* 5 of birds fed HIL-BSFLM was observed. These genera have also previously been positively correlated with poultry performance [41–44], and collectively, their short-term depletion in birds receiving high amounts of BSFLM may contribute, in part, to the poor performance exhibited for these birds. In fact, many species belonging to these genera are significant producers of SCFAs, which play a significant role in the intestinal health and growth performance of poultry. Yet, in the earlier study [18], we failed to detect any significant differences in the cecal SCFA levels between birds fed 100% BSFLM and all other control diets, suggesting that the growth-depressive effects of high amounts of BSFLM is independent of the reduced metabolic capacity of the cecal microbiota towards SCFA production.

*Romboutsia* is a genus that is commonly detected in the poultry gut [45]; however, the functional role of this genus in the intestinal tract and bird performance is currently unclear. Intriguingly, the intestinal abundance of this genus has been shown to increase in certain intestinal diseases such as dextran sulphate sodium-induced colitis in mice [46] and gastric cancer in humans [47]. Moreover, the species *Romboutsia timonensis* was first identified and isolated from the colon of patient suffering from severe anemia with melaena (gastrointestinal bleeding) [48]. To this end, a prolonged increase in the cecal abundance of *Romboutsia* in broiler chicken may serve as a bio-indicator of a compromised digestive system and, ultimately, poor growth performance.

Apart from the genus *Romboutsia*, during the grower and finisher phases, HIL-BSFLM was also associated with an increased cecal abundance of several genera including, *Bilophila*, *Intestinimonas*, *Ruminococcaceae* UCG-005/UCG-009/UCG-010, *Anaerofilum*, *Candidatus Soleaferrea*, and *Christensenellaceae R-7* group. Bacterial species belonging to several of these genera have previously been functionally linked to the health and disease status of their host including *Bilophila*, *Christensenellaceae R-7* group, *Ruminococcaceae* and *Intestinimonas* and these are discussed below. To the best of our knowledge, the genera *Anaerofilum* and *Candidatus soleaferra* are poorly described in the literature. Therefore, it is difficult to ascertain the possible impact that the enrichment of these two genera might have on poultry health and performance.

Among the *Bilophila* genus, high levels of the pathobiont *Bilophila wadsworthia* have been observed in fecal samples taken from humans suffering from Kwashiorkor, a severe form of malnutrition caused by a lack of protein [49]. Intriguingly, transplantation of the fecal flora high in *B. wadsworthia* from an individual suffering from Kwashiorkor to germ-free mice resulted in marked weight loss in recipient mice —an indication that the flora itself can directly and negatively impact body weight [49]. Additionally, transplantation of this fecal was also accompanied by perturbations in amino acid and carbohydrate metabolism in mice [49]. More recently, *B. wadsworthia* was shown to synergize with a high fat diet to promote inflammation, intestinal barrier dysfunction, bile acid and glucose dysmetabolism and hepatic steatosis in mice [50]. In 35-day-old broiler chickens, complete replacement of SBM with BSFLM was previously shown to affect amino acid metabolism [18, 19] and increase liver weight (i.e. hepatic hypertrophy) [19]. These findings are somewhat consistent with the negative effects that *B. wadsworthia* has on mouse physiology [49, 50]. Therefore, it is tempting to speculate that HIL-BSFLM-mediated expansion of *Bilophila* bacteria may interfere with broiler chicken body weight gain.

While the role of the genus *Christensenellaceae* R-7 group in poultry health is currently unclear, an inverse relationship between the relative abundance of gut *Christensenellaceae* and the body mass index of humans has previously been established [51]. Interestingly, it has been shown that amendment of an obese-associated microbiome with the species *Christensenellaceae minuta* in mice, can reduce weight gain when subsequently transferred into germ-free mice [52]. As well, a higher prevalence of *Christensenellaceae* was found in goats with lower growth rates [53]. Taken together, the *Christensenellaceae* R-7 group bacteria may have significant implications modulating body weight of broiler chicken, which may, in turn, explain the reduced performance associated with a high BSFLM content diet.

Of significance, three members of the taxonomically related members of the family *Ruminococcaceae* including UCG-005/UCG-009/UCG-010 were enriched in birds fed HIL-BSFLM. *Ruminococcaceae* family are capable of degrading and fermenting fibre containing complex polysaccharides [54] and members of this family have been reported to positively associated with poultry performance [55, 56]. Recently, Yan et al. demonstrated an increase in abundance of cecal *Ruminococcaceae* in hens fed diets supplemented with 2% and 3% BSFLM [37]. The authors of this study speculated that *Ruminococcaceae* may potentially degrade chitin (a linear polysaccharide) in BSFLM, thus explaining the increased abundance of members in this family [37]. Consistent with this, *Ruminococcaceae* were shown to increase in abundance in the human gut in response to chitin isolated from cricket powder [57]. Similarly, when frogs were switched from an herbivorous to an insect-enriched diet, *Ruminococcaceae*, which were shown to contain genes coding for putative chitin-digestion enzymes, increased in abundance within the gut [58]. These findings collectively point towards the possibility that BSFLM-derived chitin may be enriching for chitin-degrading *Ruminococcaceae* as an adaptive response to an insectivorous diet in broiler chickens.

The *Intestinimonas* genus has been shown to negatively correlate with average daily gain from days 1–48 in yellow-feathered broiler chickens [59]. Among this genus, *Intestinimonas* strain AF211 was shown to convert the Amadori product, *N*ε-fructosylysine, into butyrate [60]. *N*ε-fructosylysine is a sugar-amino acid product abundantly formed in heated foods via the non-enzymatic Maillard reaction, whereby the amino group of amino acids react with the carbonyl group of reduced sugar [60]. The Maillard reaction can cause degradation of nutritional protein quality as a result of destroying essential amino acids or reducing their availability [61], reduce protein digestibility [62], or inhibit the activity of digestive enzymes [62–64]. Importantly, commercial BSFLM undergoes common processing procedures, including heat treatment [65], which may have the potential to generate Amadori products (e.g., *N*ε-fructoselysine). Given that *Intestinomonas* was enriched in birds fed high amounts of BSFLM and that complete replacement of SBM with BSFLM in broiler chicken diets have been shown to interfere with protein digestibility [18, 20, 66], one possibility is that Maillard reaction products present in BSFLM impeded protein digestibility and this manifested in a decrease in performance and enrichment of *N*ε-fructoselysine-degrading *Intestinimonas*. Thus, future research efforts assessing the impact of heating BSFLM on the levels of Maillard reaction products warrants further investigation.

In the present study, dietary supplementation of the antibiotic BMD did not affect the overall diversity of the cecal microbiota for each phase of growth when compared to the otherwise identical diet of 0% BSFLM. In agreement with this, Proctor et al. demonstrated that the overall microbial diversity of the cecum remained unchanged in young broiler chickens fed therapeutic levels (200 g/tonne) of BMD compared to “raised without antibiotic” production [67]. Likewise, several other studies have found that the overall intestinal microbial diversity is not significantly altered by in-feed bacitracin [68–71]. Notably, *Enterococci* were found to be more abundant in chickens raised without antibiotic, regardless of the dietary exposure to BSFLM, compared to chickens fed BMD at the finisher phase (Fig. 3). This is consistent with the antibacterial activity of BMD against *Enterococci spp*., several of which are known to cause serious diseases in chickens [72]. In fact, bacitracin has previously been shown to eliminate *E. faecalis* from the intestine of broiler chickens, but not *E. faecium*, owing to an increased sensitivity of the former species to bacitracin [73]. However, the taxonomic resolution of the methods used in this study cannot discriminate between different *Enterococci* species.

In the present study, LIL-BSFLM did not impact the relative abundance of selected ARGs, indicating that inclusion of BSFLM, unlike BMD, in chicken diets has the added benefit of not enriching for ARGs important to human and animal health. This should be viewed as a positive outcome. In the ceca of birds fed BMD, the bacitracin resistance gene, *bcrR*, and the macrolide-lincosamide-streptogramin B resistance gene, *ermB*, were more abundant. In *E. faecalis*, the *bcrAB* genes encode for an ABC-type transporter (BcrAB) that is responsible for extruding bacitracin from the bacterial cell [74]. The gene *bcrD* encodes for an undecaprenol kinase, whose expression confers low levels of bacitracin resistance [75]. Expression of the *bcrABD* operon is positively regulated by the membrane-bound one-component BcrR regulator, whose gene is located upstream of this operon [76]. The gene *ermB* encodes for a ribosomal methyltransferase that dimethylates a single adenine in 23S rRNA, resulting in reduced affinity between macrolide-lincosamide-streptogramin (MLS) antibiotics and the ribosome [77]. The *bcrABDR* locus has previously been found on transferable plasmids in *E. faecalis* and *Clostridium perfringens* strains isolated from chicken samples [78, 79], as well as on the chromosome of bacitracin-resistant *C. perfringens* strains isolated from turkeys and broiler chickens [80]. Intriguingly, a multidrug resistance plasmid, pXD5, carrying both the *bcrABDR* and *ermB* genes has also been reported in *E. faecium* and *E. facecalis* of both human and swine origin [81]. This clearly indicates that bacitracin resistance and macrolide resistance can be genetically linked [81]. Given our observation that both *bcrR* and *ermB* were elevated in birds fed bacitracin, this could be evidence of BMD-mediated co-selection for bacitracin and macrolide resistance determinants.

The present study has several limitations. PCR amplification and sequencing of the V3-4 variable region of the 16S rRNA gene can introduce biases in taxonomic identification and does not provide bacterial species level resolution [82, 83]. To overcome these limitations, full-length 16S rRNA gene sequencing by PacBio and Nanopore long-read technology would have simultaneously improved the taxonomic resolution (i.e., species-level identification) of this study, while eliminating biases commonly associated with 16S rRNA gene amplicon-sequencing data [83–85]. Alternatively, a shotgun metagenomics approach would have also provided species-level resolution of the cecal microbiota [86] and would have provided insight into the potential functional metabolic capacity of the microbiota in response to dietary BSFLM. Another limitation of this study is that the bacterial microbiota of other regions along the gastrointestinal tract including the main nutrient absorption sites (duodenum, jejunum, and ileum) were not examined. Finally, while the poultry gastrointestinal tract is dominated by bacteria, other microorganisms such as fungi [87], viruses [88] and archaea [89, 90] participate in the poultry gut microbiome. Although little is known about the role of these other microorganisms in the chicken gut, elucidating the impact of dietary BSFLM as well as in-feed BMD on these microbial communities may provide insight into their performance-modulating properties.

## Conclusions

In summary, our data indicate that 12.5% or 25% BSFLM can be incorporated into broiler chicken diets with minimal impact on the cecal bacterial community and without enriching for specific antimicrobial resistance determinants. In contrast, HIL-BSFLM impacted the cecal bacterial microbiota, leading to the formation of an imbalanced microbiota that, in turn, may compromise the overall health and performance of broiler chickens. While it is unclear how the dynamic changes in the cecal microbiome contribute to BSFLM-mediated effects on broiler performance, the genera that were differentially abundant in the cecal of broiler chickens fed HIL-BSFLM may serve as biomarkers for poor growth performance. Future studies aimed at deciphering causal relationships between these candidate microbial biomarkers is crucial for a better understanding of the cecal bacterial microbiota and its function in poultry health and performance.

## Materials and methods

### Study design and sampling

Experimental details outlining bird housing, composition of experimental diets, and broiler chicken performance data have been described previously [18]. Briefly, a total of 480, day-old male Ross x Ross 708 broiler chicks obtained from a commercial hatchery (Maple Leaf Foods, New Hamburg, On, Canada) were placed in forty-eight identical metabolic cages (10 birds per cage, 8 cages per diet) and divided into 6 experimental diets in a completely randomized design. The chicks were vaccinated with Bronchitis and Marek’s vaccines at the hatchery. Mash diets were formulated for a three-phase feeding program: Starter: d 0–14, Grower: d 15–28, and Finisher: d 29–35 that met or exceeded nutrient specification for Ross x Ross 708 [91]. The diets consisted of a corn-SBM based diet with BMD (BMD group) or without BMD (0% BSFLM), and four diets where the SBM was replaced with, 12.5%, 25%, 50% and 100% commercial BSFLM (Enterra Feed Inc., Vancouver, B.C., Canada., Lot #: L191203M-1). Black soldier fly larvae were reared on a 100% plant-based diet consisting of pre-consumer food wastes.

### Sample collection

On days 14, 28, and 35, corresponding to the end of the starter, grower, and finisher phases, respectively, two birds from each cage (8 replicate cages per diet) were selected and weighed individually before being sacrificed by cervical dislocation. From each bird, ceca were aseptically collected and transferred into a sterile Whirl-Pak plastic bag (Nasco, Fort Atkinson, WI, USA), immediately placed on dry ice for transportation, and subsequently stored frozen at -80°C until further analysis. Day 35 broiler chicken excreta was also collected aseptically from slide-in trays that were placed underneath each of the 48 wire-mesh-floor cages and stored at -20°C for later analysis.

### Microbial DNA isolation

Total microbial genomic DNA was extracted from 250 mg of cecal digesta and litter samples using the QIAmp Power Fecal Kit Pro DNA kit (Qiagen, Toronto, ON, Canada) and the DNeasy PowerSoil Pro Kit (Qiagen, Canada), respectively, according to the manufacturer’s instructions and using a final elution volume of 100 µl. In several instances, when the cecal digesta available was less than 250 mg, the entire digesta sample (40–190 mg) was used for extracting microbial DNA with the elution volume adjusted accordingly. Unless otherwise specified, the quality of the extracted nucleic acid samples were individually assessed using a Nanodrop™ One/One^C^ Microvolume UV-Vis Spectrophotometer (Thermo Fisher Scientific, Toronto, ON, Canada) (A260/280) and by agarose gel-electrophoresis. The DNA concentrations of samples were determined using the Qubit double-stranded DNA high-sensitivity assay kit with a Qubit 4.0 Fluorometer (Thermo Fisher Scientific, Canada).

### Microbial profiling 16S rRNA gene sequencing

To determine the composition of the cecal microbial communities, an Illumina 16S metagenomics sequencing workflow (Illumina Canada, Vancouver, BC, Canada) targeting the V3 and V4 variable region of the 16S rRNA gene was employed [92]. Briefly, the V3 and V4 region of the 16S rRNA gene (~ 460 bp) was amplified from the cecum-derived microbial genomic DNA in a 25 µl PCR mixture containing 12.5 ng of template DNA, 200 nM of each of the 16S amplicon PCR forward and reverse primers, and 1 x KAPA HiFi Hot start Ready Mix (Roche, Millipore-Sigma, Mississauga, ON). The PCR mixtures were heated for 3 min at 95^o^C, followed by 25 cycles of 30 s at 95°C, 30 s at 55°C, 30 s at 72°C, before finishing with 5 min at 72°C. The 16S rRNA gene amplicons were purified using AMPure XP magnetic beads (Beckman Coulter, Mississauga, ON, Canada), and adaptor-incorporated unique dual-indices were added to the amplicons using the Nextera XT index Kit v2 (Illumina, Canada) in accordance with the Illumina protocol. Indexed amplicon libraries were then further purified and normalized using the NGS normalization 96-well kit (Norgen Biotek, Thorold, ON, Canada) before pooling to a final concentration of 4 nM. High-throughput sequencing was performed on a MiSeq sequencer (Illumina, Canada) using the MiSeq v3 kit (600-cycle) and a loading concentration of 8 pM with 10% PhiX spike-in, to generate 2 × 300 bp paired-end sequences, targeting an output of 100,000 raw reads/sample.

### Bioinformatics analyses

QIIME2, an end-to-end bioinformatics pipeline, with custom settings and the DADA2 option chosen for denoising, was used for downstream processing of the 16S rRNA gene sequencing output [93]. To assign taxonomy to the amplicon sequence variants, SILVA SSU reference database (release 132) was used for training classifiers. To remove primer sequences and any low-quality bases at the 5’ end, the first 21 bases from the forward read and the first 21 bases from the reverse-read were discarded. For quality trimming at the 3’ end, the forward and reverse reads were truncated at position 280 and 219, respectively, based on manual quality inspection.

### Detection and quantification of antimicrobial resistance-associated gene targets

The abundance of 14 selected gene targets associated with antibiotic resistance (*sul1*, *strA*, *strB*, *blaOXA-20*, *ermB*, *ermF*, *aadA*, *blaCMY-2*, *tetA*, *sul2*, *mphE*, *msrE* and *bcrR*) or horizontal gene transfer (*int1*), were determined by quantitative real-time PCR (qPCR) using a Bio-Rad CFX96 real-time PCR instrument (Bio-Rad Laboratories, Mississauga, ON, Canada) with Bio-Rad CFX Maestro software version 3.0, as previously described [94]. The relative abundance of each gene was determined as the ratio of targeted gene copy number per total 16S rRNA gene, *rrnS*, copy numbers in the reaction. The primers and hydrolysis probes used in the present study are listed in Additional file 1, Table S3. All primers and hydrolysis probes were synthesized by Sigma (Sigma–Aldrich, Toronto, ON, Canada). The qPCR assays were performed using the Brilliant II QPCR Master Mix (Agilent) for TaqMan PCR and the Brilliant II SYBR Green® Low ROX qPCR Master Mix (Agilent, Toronto, ON, Canada) for SYBR Green qPCR. Two microlitres of template DNA (corresponding to 0.1–10 ng DNA) were added to each qPCR reaction mixture to reach a final volume of 25 µl. Each sample reaction, including the template-free control reaction, was run in triplicate with the following cycle conditions: 1 cycle at 95°C for 10 min, followed by 40 cycles of 95°C for 15 s, and the established annealing temperature and extension time as specified for each primer set in Additional file 1. For the SYBR Green assay, melt curve analyses was performed as previously described [94]. When using TaqMan chemistry, the identity of the quantified gene targets were ensured on the basis of hybridization. The expected PCR product for each target gene was synthesized and subcloned into the vector, pBlueScript II SK (+)  (Bio Basic Inc., Markham, Ontario, Canada). Plasmid copy numbers were calculated using the measured DNA concentration from a NanoDrop N-1000 spectrophotometer. Both amplification efficiencies (i.e., acceptable amplification efficiency between 95 and 105%) and gene target copy numbers were determined using standard curves consisting of a 10-fold serial dilution of a known target plasmid (from 10^7^ to 100 copies/µl). Gene targets that were detected at copy numbers below 1–4 copies per reaction were considered to be below the limit of quantification [94].

### Statistical analysis and data visualization

Unless otherwise specified, microbial compositional analysis was conducted using the open-source program R and RStudio (ver. 1.4.1106) [95]. The QIIME2-derived taxonomic assignment outputs were processed using R-based microbiome analytical packages *qiime2R* ver. 0.99.4 [96] and phyloseq ver. 1.34.0 [97], before graphical visualization by *ggplot2* ver. 3.3.3 [98]. Contaminating sequences from negative control samples were identified and removed using the “ prevalence method” of the R *decontam* package [99]. To estimate alpha diversity, diversity indices were computed using R package *vegan* ver. 2.5-7 [100] and the statistical tests of non-parametric Kruskal-Wallis test and pairwise Dunn’s post-hoc test, with bonferroni correction to adjust *p* values for multiple pairwise comparisons, performed using the functions “kruskal_test” and “dunn-test” from R package *rstatix* ver. 0.7.0 [101]. Beta-diversity was estimated by performing principal coordinate analysis (PCoA) based on the Bray-Curtis dissimilarity using the “vegdist” and “pcoa” functions of R packages *vegan* and *ape* ver. 5.4-1 [102]. Significance of dissimilarity between groups were examined by permutational multivariate analysis of variance (PERMANOVA) using *vegan* function “adonis2” with 999 permutation, while the statistical significance of difference detected between any two groups was determined using the function “pairwise adonis” of R package *pairwiseAdonis* ver. 0.4 [103] with the Bonferroni method chosen for error correction and 999 permutations. The differential abundances of individual taxa between groups associated with different diets were determined using the Analysis of Compositions of Microbiomes with Bias Correction (ANCOM-BC2) methodology [32], conducted by running the “ancombc2” function of R package *ANCOMBC* ver. 2.02 with default options to take into account the independent variables of diet and cage separation as fixed- and random-effect, respectively. Any differential abundance with statistical significance at the bacterial taxonomic ranks of genus based on the mixed-directional false discovery rate-controlled Dunnett’s type of test output of ANCOM-BC2 were reported as log fold changes (with standard errors) relative to the reference BMD diet group. Non-parametric Mann Whitney U tests were used to compare antibiotic resistance-associated gene abundance between BSFLM-fed birds and BMD-fed birds, using GraphPad Prism v9.2 (La Jolla, CA, United States).

### Electronic supplementary material

Below is the link to the electronic supplementary material.

**Additional file 1: Supplementary Material 1. Table S1.** 16S rRNA gene sequencing output statistics derived from QIIME2-enabled DADA2 plugin; **Table S2.** Two-factor permutational analysis of variance (PERMANOVA) results for the broiler chicken cecal bacterial community composition; **Table S3.** Primers and probes used for quantitative PCR in detection of antimicrobial resistance genes.

**Additional file 2: Supplementary Material 2. Figure S1.** Box and whisker plots comparing the effects of age on the alpha diversity metrics of the cecal bacterial community for a given diet; **Figure S2.** Genus-level PCoA based on the Bray-Curtis dissimilarity comparing the effects of age on the cecal bacterial community composition of chickens fed different experimental diets; **Figure S3.** Genus-level PCoA based on the Bray-Curtis dissimilarity comparing the effects of diet on the cecal bacterial community composition of chickens at each growth phase.

## Data Availability

All biological sequence data are accessible on the NCBI server under BioProject identifier (ID)PRJNA998860. Nucleotide sequences for 16S rRNA amplicon sequence data were submitted to the Sequence Read Archive (SRA) under SAMN36715391–SAMN36715686. All other data generated or analyzed during this study are included in this published article and its supplementary information files.
