# Supplementary material for: Dynamic effects of black soldier fly larvae meal on the cecal bacterial microbiota and prevalence of selected antimicrobial resistant determinants in broiler chickens
Source: Anim Microbiome. 2024 Feb 15;6:6. doi: 10.1186/s42523-024-00293-9 (PMC10868003; doi:10.1186/s42523-024-00293-9)
Supplement: Supplementary file 2 — Additional file 2: Supplementary Material 2. Figure S1. Box and whisker plots comparing the effects of age on the alpha diversity metrics of the cecal bacterial community for a given diet; Figure S2. Genus-level PCoA based on the Bray-Curtis dissimilarity comparing the effects of age on the cecal bacterial community composition of chickens fed different experimental diets; Figure S3. Genus-level PCoA based on the Bray-Curtis dissimilarity comparing the effects of diet on the cecal bacterial community composition of chickens at each growth phase. [file 42523_2024_293_MOESM2_ESM.docx]

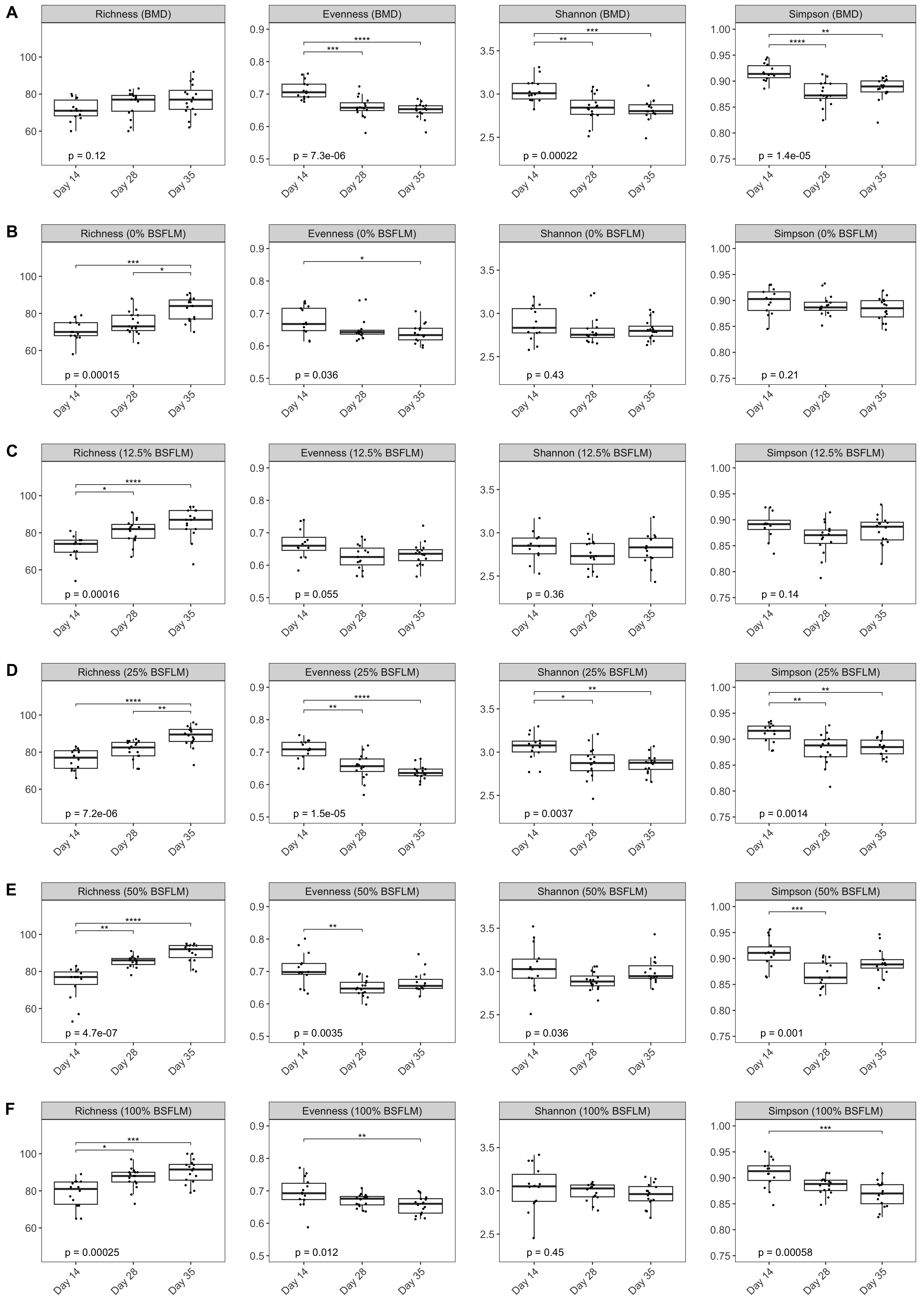


**Supplemental Figure S1.** Alpha diversity metrics of cecal microbiota at each growth phase for broiler chickens fed BMD (A), 0% BSFLM (B), 12.5% BSFLM (C), 25% BSFLM (D), 50% BSFLM (E), and 100% BSFLM (F). The box-and-whisker plots show the first and third quartile (bottom and top lines of the box) and the median (horizontal middle line) values of the indicated diversity metrics. Each broiler chicken cecal sample is represented by a solid black dot. Statistical significance of between-group differences were detected using the non-parametric Kruskal-Wallis test followed by post-hoc pairwise Dunn test with Bonferroni correction and are indicated with an asterisks as follows: * *p* < 0.05; ** *p* < 0.01; *** *p* < 0.001; **** *p* < 0.0001. BMD, bacitracin methylene disalicylate; BSFLM, black solider fly larvae meal; %, percentage of soybean meal replaced with black soldier fly larvae meal (BSFLM).


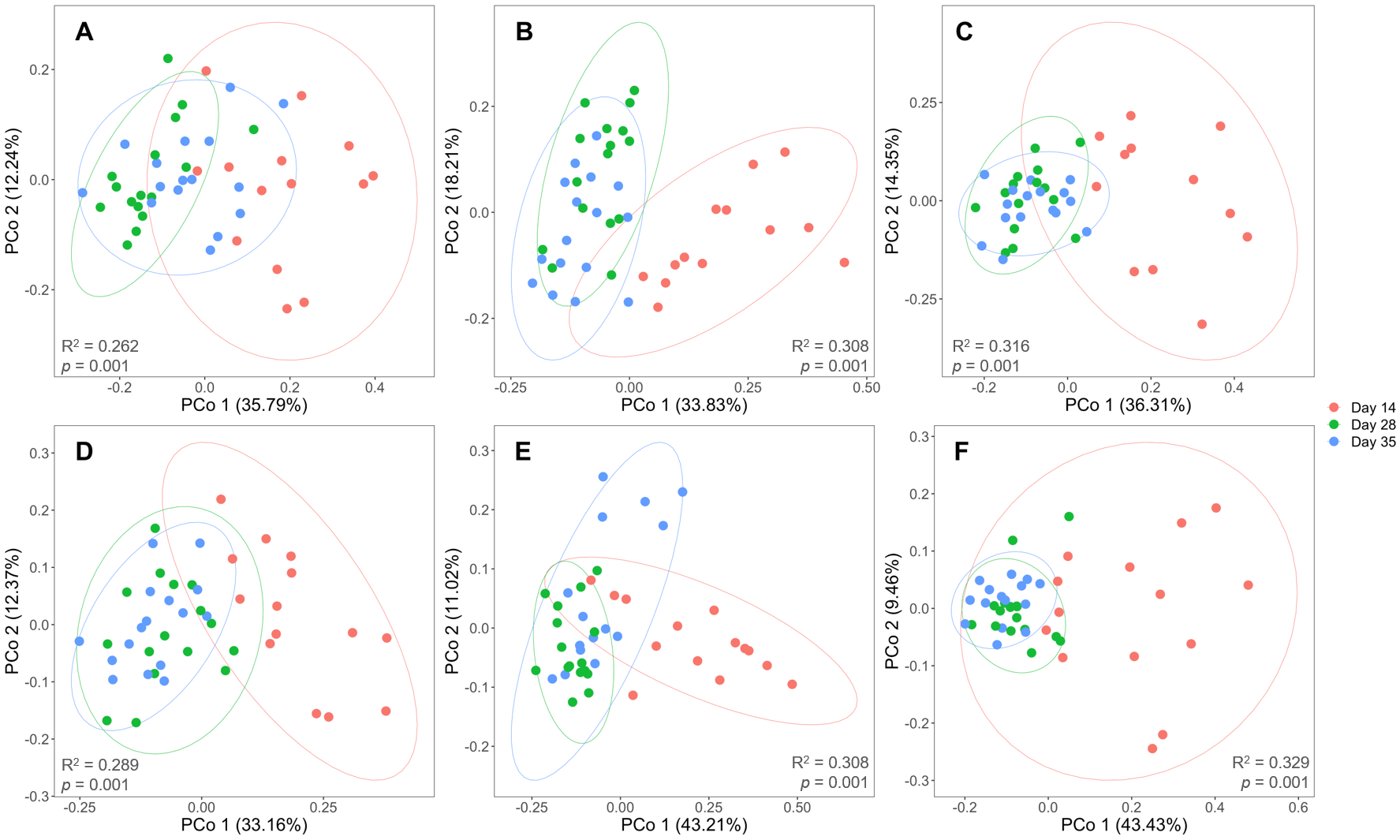


**Supplemental Figure S2.** Genus-level principal coordinates analysis based on the Bray-Curtis dissimilarity comparing the impact of age on the cecal bacterial community of birds fed BMD (A), 0% BSFLM (B), 12.5% BSFLM (C), 25% BSFLM (D), 50 % BSFLM (E) and 100% BSFLM (F). Percentages of total variance explained by each principal coordinate (PCo1 and PCo2) are displayed in the axis titles. Colored solid dots represent broiler chicken cecal samples at different growth phases (red, day 14; green, day 28; blue, day 35). Colored ellipses correspond to the 95% confidence intervals for each growth phase.


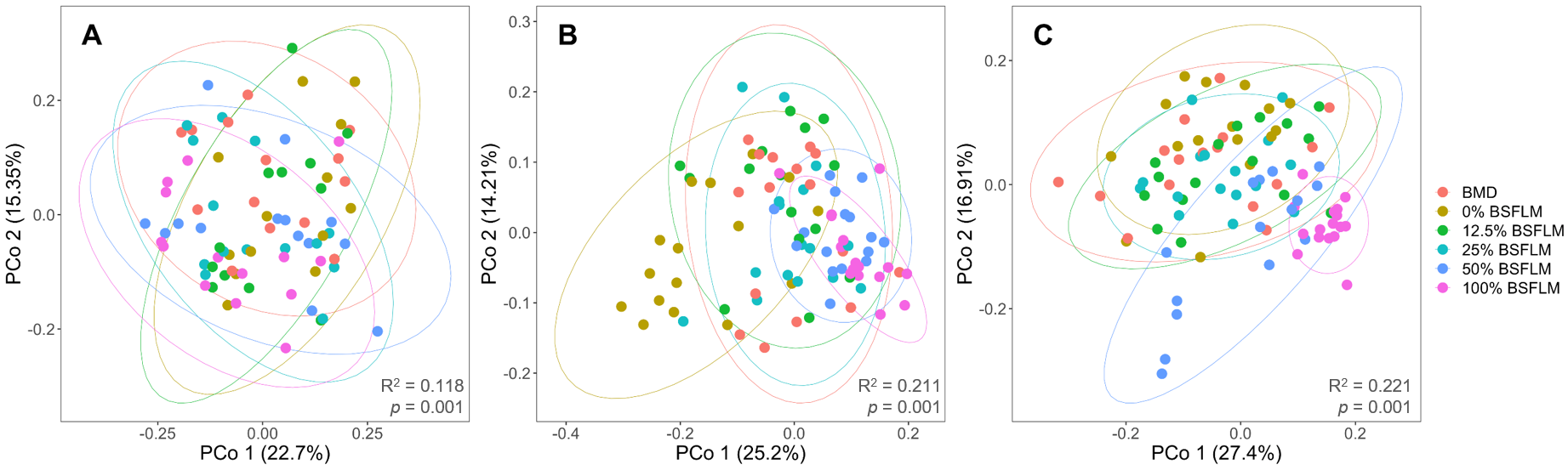


**Supplemental Figure S3.** Genus-level principal coordinates analysis based on the Bray-Curtis dissimilarity comparing the impact of diet on the cecal bacterial community at the end of day 14 (A), day 28 (B) and day 35 (C). Percentages of total variance explained by each principal coordinate (PCo1 and PCo2) are displayed in the axis titles. Colored solid dots represent broiler chicken cecal samples from each dietary group (red, BMD; brown, 0%BSFLM; green, 12.5% BSFLM; teal, 25% BSFLM; blue, 50% BSFLM; pink, 100% BSFLM). Colored ellipses correspond to the 95% confidence intervals for each growth phase.
